# Design of an interactive brain model for neuroanatomy education and MRI training

**DOI:** 10.1002/ase.70009

**Published:** 2025-02-27

**Authors:** Ethan P. McNaughton, Liam Bilbie, Matea Zuljevic, Lauren K. Allen, Daiana‐Roxana Pur, Roy Eagleson, Sandrine de Ribaupierre

**Affiliations:** ^1^ Schulich School of Medicine & Dentistry Western University London Ontario Canada; ^2^ Department of Electrical & Software Engineering, Faculty of Engineering Western University London Ontario Canada; ^3^ Department of Clinical Neurological Sciences, Schulich School of Medicine & Dentistry Western University London Ontario Canada; ^4^ Department of Anatomy & Cell Biology, Schulich School of Medicine & Dentistry Western University London Ontario Canada

**Keywords:** computers in anatomical education, interactive computer graphics, modeling and simulation, teaching of neuroscience/neuroanatomy

## Abstract

In this article, we introduce a new virtual application that offers an interactive model of the brain for neuroanatomy education. Through a dual‐platform architecture, the application can be downloaded on both desktop and mobile devices, with the mobile app leveraging unique capacities of modern handheld systems to deploy the brain model in augmented reality. In addition to illustrating complex spatial relationships between internal brain structures, vasculature, and cranial nerves, the application integrates magnetic resonance imaging (MRI) data into the user interface. MRI series in the coronal, sagittal, and axial planes can be superimposed directly onto the brain model, allowing students to engage with two‐dimensional MRI slices in three‐dimensional space. While previous virtual tools have offered a similar superimposition, none have done so through a mobile app, downloadable on handheld devices and suited to the modern student. The benefits of this function on students' spatial understanding and identification of neural structures on MRI slices remain understudied. The aim of this article is to describe the functionality of our dual‐platform application, to outline its potential strengths as an educational tool, and to address possible directions for improvement following future assessments of the app's utility. Our ultimate goal is to offer a preliminary introduction to a new system that seeks to support users' understanding of three‐dimensional neuroanatomy and aims to enhance their ability to read an MRI of the brain.

## INTRODUCTION

Anatomy's pedagogy has undergone drastic change in the modern world. Early studies warned of a decrease in the time and resources allocated to teaching the subject,^1^, ^2^, ^3^ leading students and educators alike to express concerns for future clinicians' ill‐preparedness for professional practice.^4^, ^5^ While the time dedicated to lecture material has marginally rebounded in recent years, hours spent in a laboratory environment have continued to decrease.^6^

Amidst a transformative period for anatomy education, the subject's pedagogy continues to be critically evaluated by researchers.^3^, ^7^, ^8^, ^9^, ^10^ Human body dissection has long constituted the “gold standard” for teaching anatomy,^9^, ^11^ and it is still widely seen as an important means of preparing students for clinical work.^5^, ^12^, ^13^ However, the increasing costs and declining availability of body donors in educational settings are notable drawbacks.^14^, ^15^, ^16^, ^17^ Physical three‐dimensional (3D) models have been proposed as a solution to these issues while still offering students the opportunity for tactile manipulation^18^ and observation through stereoscopic vision.^19^, ^20^ However, these models are commonly criticized for their inability to replicate the fine details of true anatomical structures, thus calling into question their capacity to transfer knowledge from artificial to authentic anatomy.^21^, ^22^, ^23^ Technology's potential to improve the current pedagogical landscape has gained considerable interest in recent years.^17^, ^24^ Virtual tools—especially those accessible on handheld mobile devices—allow students to engage in content at a time, location, and speed that best suits themselves, while educators can provide resources of higher quality and quantity that curate a customized learning experience.^25^, ^26^, ^27^, ^28^, ^29^

Augmented reality (AR) offers an immersive virtual setting for users to interact with pseudo‐3D models on their mobile devices, giving the technology great potential in anatomy education.^30^, ^31^ Anatomy students report high levels of satisfaction using a variety of distinct AR tools in a learning environment.^32^, ^33^, ^34^, ^35^ AR boosts student motivation to facilitate both increased engagement in learning and improvements to academic performance on written anatomy tests.^35^, ^36^, ^37^, ^38^ Elevated test scores are also linked to students' enhanced spatial understanding using AR, with users demonstrating a greater proficiency in conceptualizing relative locations in 3D space than control groups.^37^, ^39^, ^40^ Anatomy requires a great deal of cognitive processing power for students, especially when attempting to grasp 3D information from 2D diagrams.^41^, ^42^ AR effectively reduces students' cognitive load to enhance anatomy exam performance.^38^ Because the brain's structure tends to be a particularly intimidating component of students' education,^43^, ^44^, ^45^ it is notable that these findings have been replicated in the subdomain of neuroanatomy. AR's enhancement of spatial understanding^46^ and its diminishment of cognitive load^47^ show its potential to improve neuroanatomy knowledge acquisition.^48^, ^49^

With the recent adoption of a multi‐modal approach to anatomy education,^8^, ^10^, ^15^, ^50^ it is vital that technological development aligns with the pedagogical strengths of virtual tools. Unlike the traditional methods of learning anatomy, virtual resources host the ability to expose students to technologies widely used in research and healthcare settings. A primary example of such technologies is magnetic resonance imaging (MRI). Practitioners must seamlessly and bidirectionally associate neural structures with their appearance in two‐dimensional (2D) MRI slices. In an effort to support student learning of the spatial relations between 3D neuroanatomy and 2D cross‐sectional images, past virtual tools have superimposed MRI slices onto 3D models.^42^, ^51^, ^52^ However, to our team's knowledge, none have done so through a mobile app—a medium that is suited to the modern student, free of cost, and easily implemented both within the classroom and independent of institutional education. It is the view of our research team that a superimposition of MRI series onto a virtual anatomy model accessible via widespread handheld devices may benefit students' neuroanatomy education and ability to identify neural structures in MRI slices. Furthermore, the integration of healthcare‐based technologies with mobile‐phone‐based teaching tools may better facilitate the translation of students' established academic improvements using AR^35^, ^36^, ^37^, ^38^ into clinical success in patient care. Nonetheless, the pedagogy of reading MRI slices is an understudied field, and it remains unclear what benefits such an integrated approach might have on student learning.

Our research team had previously developed a 3D model of the brain in virtual reality (VR), accessible to students via desktop.^48^ Further development has since been focused in two primary directions: (1) the construction of a complementary mobile app presenting an identical anatomy model in augmented reality, and (2) the integration of MRI data into the application's dual‐platform system. These updates open up new opportunities to compare virtual and augmented realities in neuroanatomy education and to further elucidate the educational benefits of MRI superimposition onto virtual anatomy models. While empirical assessment of the model's AR and VR components is thus needed, in this article, we offer a preliminary introduction to the app, outline its construction, and describe the functionality of its two primary technical developments.

## DESCRIPTION

### Construction

Our research team's original VR project acted as the starting point for the current model. This project had obtained 1.5 Tesla MRI data (taken at 4 mm intervals at a resolution of 256 pixels by 256 pixels) from the open‐source data sets of the Visible Human Project (VHP), released by the National Library of Medicine (NLM).^53^ Female brain scans were manually segmented in FMRIB Software Library^54^ (4.1), and volumetric rendering was completed using Visage Imaging's Amira (5.1) software package.^55^ The development of our team's original VR model is outlined in greater detail in previous work.^48^, ^49^

The mobile application was developed with Unity 3D^56^ (2020.3.33f1). The model was packaged from Blender^57^ (2.83) and edited using Unity's AR Foundation^58^ (5.0.7), a framework for developing platform‐agnostic AR applications. AR Foundation's plane detection was used to identify horizontal planes in users' external environments, allowing model deployment and raycast translation. Further app functionality, including model rotation, isolation and description of neural substructures, and quiz construction were completed with the various development tools available in the framework supplied by Unity's AR Foundation.

The application was made deployable on both Android and iOS devices using a model‐driven approach to software development.^59^ Android build files (.apk files) were generated directly in Unity, while the iOS build outputs ran as XCode^60^ projects and were deployed using TestFlight.^61^ Open access to the application on Android's Google Play Store and iOS' App Store awaits prospective results of a comprehensive study on the app's utility to ensure the released product is verifiably effective in supporting student learning.

### Functionality

**FIGURE 1:**
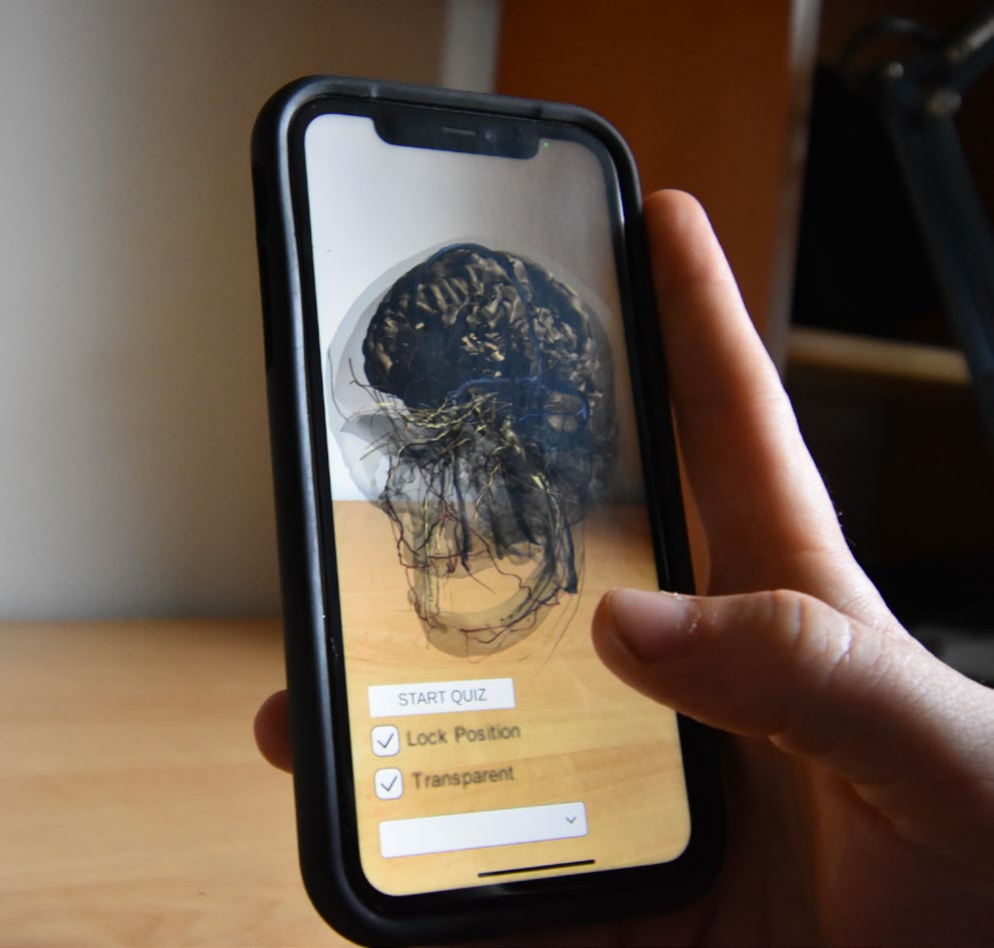
Users' point of view upon model deployment in the mobile app. The AR model is virtually placed in the user's external environment. It can be placed, resized, and rotated by the intuitive controls of tapping, pinching, and swiping. The “Lock Position” checkbox ensures that the model remains stationary in external space, regardless of camera movement.

Upon installation, users are prompted to scan their local environment for the detection of horizontal planes. With the camera pointed at users' desired location of placement, tapping on the device's screen deploys the model. Users may pinch to adjust the model's size before locking it in place using the “Lock Position” checkbox. Swiping a finger horizontally rotates the model to permit a 360 degree view of the head's anatomy. Figure 1 illustrates a user's view upon opening the app and deploying the model.

**FIGURE 2:**
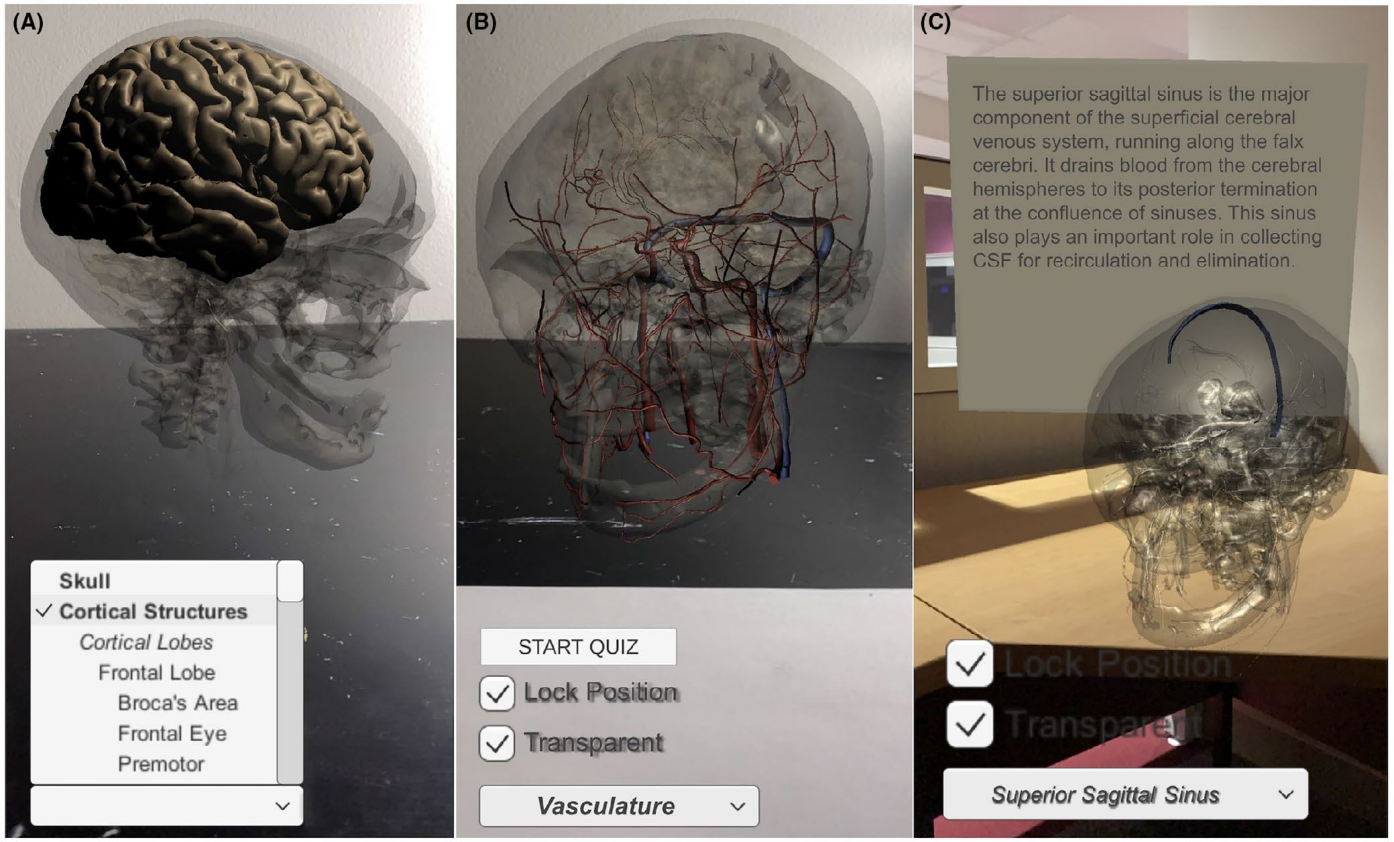
Screenshots of application functionality as seen within the mobile platform. (A) Drop‐down menu. Users can scroll through the model's components to learn more about each. (B) Visual isolation of a single structural component. Using the “Transparent” checkbox, the model is faded to heighten the visibility of the brain's vasculature. (C) Functional descriptions of selected structures. Upon selecting the superior sagittal sinus, a floating text box remains stationary in the user's external space to outline the sinus' functional role.

On both the mobile and desktop platforms, users can comprehensively explore the anatomy of the brain and its associated systems. Included structures of the central nervous system are the cortical lobes and their sub‐regions, the deep neural and ventricular systems and their associated areas, and the cerebellum. The model also offers users an in‐depth view of the brain's associated systems, including the vasculature and its constituent components, the cranial nerves, and the dural venous sinuses. Anatomical structures can be highlighted using a drop‐down menu in the corner of users' screens (see Figure 2A). Users can subsequently choose to study the structure's spatial location relative to other neural structures, or instead isolate the structure by fading all other regions using the “Transparent” checkbox (see Figure 2B). On top of the model's structural information, functional roles of each anatomical component are outlined (see Figure 2C).

Users can engage in self‐assessment using the app's quiz function. Tapping the “Start Quiz” button launches a 10‐question multiple‐choice quiz. Each question randomly highlights a structure within the model and offers four potential answers. After completion, students can review feedback on their score and time taken before returning to the home page.

**FIGURE 3:**
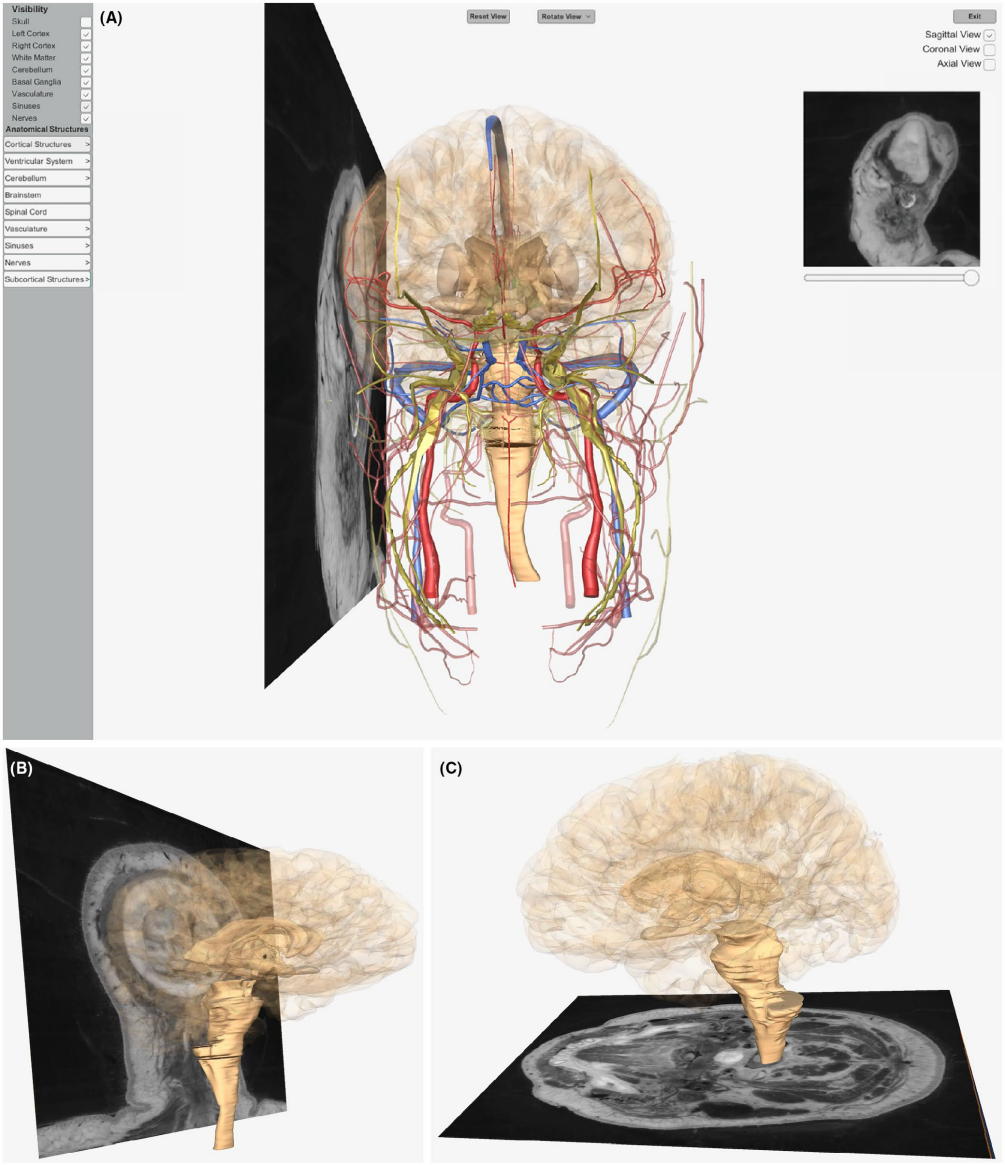
Screenshots of MRI superimposition as seen in the desktop platform. (A) Users' view upon deploying the MRI feature. Clicking the “Sagittal View” checkbox superimposes MRI slices onto the model in the sagittal plane. Dragging the scrollbar translates the slices along the model to align with its 3D components and adjusts the slice preview (top‐right) accordingly. (B) Close‐up of coronal MRI slice deployment. (C) Close‐up of axial MRI slice deployment.

MRI data, retrieved from the same NLM VHP dataset used to construct the model,^53^ were integrated into the application to assist students in translating their 3D neuroanatomy knowledge to the brain's appearance in widely used 2D images. Figure 3 exhibits this feature as it appears in the application's desktop‐based platform. 2D MRI slices are overlaid in alignment with their corresponding planes within the 3D model. To demonstrate how the model's anatomical structures appear in an isolated MRI view, the slices can be previewed in the top‐right corner of users' screens. Dragging a scrollbar translates the slice across the model perpendicular to the chosen plane, adjusting the slice preview accordingly. With 75 images accessible across the three spatial planes, users gain comprehensive exposure to MRI data in the sagittal, coronal, and axial views. Slices overlaid onto the model in each of these planes are depicted by Figure 3A–C, respectively.

### Pilot study

To gain an early indication of the app's utility as an effective educational tool, 12 anatomy students at the University of Western Ontario were recruited to participate in a survey on the virtual brain model's functionality. The app received an average rating of 4.67 out of 5 across the 12 study participants on its “usefulness”, while 11 of the 12 students agreed that the app was “immersive” and “conducive to [their] learning”. However, with the opportunity to provide their open feedback, two students expressed difficulty in viewing small structures (e.g., the pontine arteries). Ultimately, 11 of the 12 students agreed that the app would be a useful tool for assisting them in studying neuroanatomy, each reporting that they would be likely to recommend the app to their peer students. The results of this survey act as preliminary indicators of the application's utility, but further testing is needed for a more comprehensive and rigorous assessment.

## DISCUSSION

As a virtual resource widely accessible on both desktop and mobile devices, our application overcomes many of the barriers faced by traditional teaching methods. Unlike body donor dissection, the app exposes students to the intricacies of neuroanatomy free of cost. It eliminates the financial and accessibility issues associated with human body dissection, as well as its necessity for lab‐based curricular rigidity.^9^ Students can engage in educational content at a time, place, and rate that best suit their individual needs, allowing the app to be easily implemented into large class learning environments without sacrificing its personalizable character. While physical 3D models largely share these advantages, the app is capable of replicating authentic anatomy more realistically than its physical counterparts.^23^ Most importantly, our system integrates 3D anatomy with 2D imaging techniques, offering a pedagogical benefit only available through virtual resources. With this technological novelty, our application is expected to align with meta‐analyses indicating virtual tools' capacity to boost student motivation and improve neuroanatomy comprehension.^39^, ^62^ While traditional learning methods remain important for the teaching of neuroanatomy,^8^, ^10^, ^50^ the application shows early promise as a strong complementary tool to the widely utilized methods of education.

Previously developed virtual platforms have similarly superimposed MRI data onto 3D neuroanatomical models, but have not done so through a mobile app accessible on handheld devices. Computer‐based,^42^ video‐based,^51^ and headset‐based^52^ resources have integrated 2D slices into 3D space, effectively illustrating neuroanatomical spatial relations across these visualization formats. Simultaneously, a number of mobile applications have been developed to assist students' neuroanatomy education, including GreyMapp‐AR,^47^ Brain Tutor 3D,^63^ Head Atlas,^64^ and VH Dissector.^65^ However, to the research team's knowledge, no other platform has superimposed MRI slices onto a 3D neuroanatomical model in a mobile app setting, allowing for this function to be made maximally accessible to students using handheld devices.

Despite the potential for the current app to support student learning, two limitations have become apparent. As indicated by feedback in the pilot study, future app iterations may improve on the visibility of small anatomical structures. The inclusion of an automatic zoom function during user interactions with these minute components is predicted to further reinforce the application's efficacy for clearly depicting the brain's anatomy. Furthermore, the application's quiz function may be improved through individual customization. Personalized educational activities targeted at identifying and improving upon areas of weakness best support student success.^66^, ^67^ Future versions of the app may therefore build upon the existing quiz function by longitudinally saving students' quiz results within the app's framework. With a measure of individual progress, subsequent quizzes can personalize their content focus.

The benefits of superimposing MRI data directly onto 3D anatomical models remains an understudied field, but a preliminary assessment indicated its potential utility in facilitating student learning. Using “a combined model in which 3D objects are overlaid onto the 2D MRI slices,” the study showed that students using an integrated approach achieved higher comprehension scores than those engaging with 3D models and 2D slices separately.^42^ This effect was particularly significant for the teaching of C‐shaped internal brain structures including the lateral ventricles, caudate, fornix, and hippocampus. It is our hope that a comprehensive follow‐up study evaluating our developed application will further elucidate the potential educational benefit of MRI superimposition onto 3D learning resources. Through this evaluation, we additionally hope to compare the utility of our mobile app with our previously‐constructed VR model to understand the relative effectiveness of AR and VR in anatomy education.

## AUTHOR CONTRIBUTIONS

**Ethan P. McNaughton:** Writing – original draft; writing – review and editing; conceptualization; methodology. **Liam Bilbie:** Software; methodology; conceptualization. **Matea Zuljevic:** Conceptualization; writing – review and editing; resources. **Lauren K. Allen:** Conceptualization; resources. **Daiana‐Roxana Pur:** Conceptualization. **Roy Eagleson:** Conceptualizaion; Writing – review and editing; project administration; supervision; validation. **Sandrine de Ribaupierre:** Conceptualization. Writing – review and editing; project administration; supervision; validation.

## CONFLICT OF INTEREST STATEMENT

The authors have no conflicts of interest to declare in this research.
